# Fecal Microbiota Transplantation as a Novel Therapy for Ulcerative Colitis: A Systematic Review and Meta-Analysis

**DOI:** 10.1097/MD.0000000000003765

**Published:** 2016-06-10

**Authors:** Dali Sun, Weiming Li, Shumin Li, Yunyun Cen, Qingwen Xu, Yijun Li, Yanbo Sun, Yuxing Qi, Yueying Lin, Ting Yang, Pengyuan Xu, Qiping Lu

**Affiliations:** From the Department of Gastrointestinal Surgery (SD, LW, LS, CY,XQ, LY, SY,QY, LY, YT,XP), Second Affiliated Hospital of Kunming Medical University; Research Center for Surgical Clinical Nutrition in Yunn an Province (SD, LW, LS, CY,XQ, LY, SY,QY, LY, YT, XP), Kunming; and Department of General Surgery (SD, LQ), Wuhan Clinical School, Southern Medical University, Wuhan, China.

## Abstract

Variation in clinical evidence has prevented the adoption of fecal microbiota transplantation (FMT) in patients with ulcerative colitis (UC). We aimed to conduct a systematic review and meta-analysis to determine the efficacy and safety of FMT in UC.

A systematic literature search was performed in 5 electronic databases from inception through September 2015. Inclusion criteria were reports of FMT in patients with UC. Studies were excluded if they did not report clinical outcomes or included patients with infections. Clinical remission (CR) was defined as the primary outcome.

Eleven studies (2 randomized controlled trials (RCTs), 1 open-label case-control study, and 8 cohort studies) with a total of 133 UC patients were included in the analysis. In 11 studies (including 8 noncontrol cohort studies and the treatment arms of 3 clinical control trials), the pooled proportion of patients who achieved CR was 30.4% (95% CI 22.6–39.4%), with a low risk of heterogeneity (Cochran *Q* test, *P* = 0.139; *I*^2^ = 33%). A subgroup analysis suggested that no difference in CR was detected between upper gastrointestinal delivery versus lower gastrointestinal delivery. Furthermore, subgroup analysis revealed that there was no difference in CR between single infusion versus multiple infusions (>1) of FMT. All studies reported mild adverse events.

FMT is potentially useful in UC disease management but better-designed RCTs are still required to confirm our findings before wide adoption of FMT is suggested. Additionally, basic guidelines are needed imminently to identify the right patient population and to standardize the process of FMT.

## INTRODUCTION

Ulcerative colitis (UC) is a chronic, relapsing, and remitting disease characterized by inflammation of the colonic mucosa. UC is a subtype of inflammatory bowel disease (IBD) causing significant morbidity. Epidemiological studies have shown a significant increase in the incidence of UC across the world (about 3 million of people).^1,2^ While the precise etiology of UC remains unclear, several risk factors including immunologic, genetic, environmental, and gut microbial have been proposed. Several studies have suggested that gastrointestinal microbiota might play a role in development of this disease.^3^ Specifically, microbial dysbiosis has been hypothesized as a trigger in UC disease development.^4^

Treatments that manipulate the microbiota balance have been developed and investigated including the administration of probiotics and prebiotics, with different evidences observed for their efficacy.^5,6^ An additional alternative therapy for the management of UC is fecal microbiota transplantation (FMT), which has been shown as an effective treatment for refractory and recurrent *Clostridium difficile* infection (CDI).^7^ The success of FMT in treating *C difficile* infections has raised the possibility that FMT may be beneficial in other diseases through alterations in gastrointestinal microbiota or dysbiosis.

The use of FMT in UC patients was first described by Bennet in 1989.^8^ In this case, Bennet treated himself, an active and severe UC patient, using FMT and he was symptom-free for 6 months subsequently. Afterward, other reports were published, most of which were case reports or noncontrol cohort studies, with variable results. Recently, some new cohort studies^9–13^ and the first 2 randomized, double-blinded, controlled trials^14,15^ were presented, but the findings in these studies are variable which has unfortunately confused UC clinicians.

Only 1 meta-analysis of IBD included both unpublished and abstract data including 1 abstract of RCT.^16^ Additionally only 4 noncontrol cohort studies of UC patients with FMT (27 cases) were performed in a subgroup analysis that showed a pooled estimate for achieving remission of 24.1% (95% CI 11.1–44.9%). The validity of that data was limited by the methodological concerns and the lack of an adequate number of studies.^16^ Three systematic reviews of IBD also included some case reports and noncontrol cohort studies of UC patients,^17–19^ but also contained several methodological limitations. In fact, 2 systematic reviews did not analyze the subgroup of UC,^17,18^ while 1 systematic review contained mostly case reports and FMT outcomes that were measured by treatment “success rates” and not by any other more validated measures.^19^

The aim of this study was to undertake a systematic review and meta-analysis of FMT in patients with UC so as to provide clinicians with a comprehensive and clear assessment of the available evidence upon which to guide current practice and future research.

## METHODS

### Search Strategy

We followed the MOOSE, PRISMA, and Cochrane guidelines in our study.^20–22^ An electronic search was conducted using PubMed, Cochrane Library, Web of Science, Wanfang Data, and China National Knowledge Infrastructure. All databases were searched from their inception through September 2015. No language limits were used. Searching was limited to publications with clinical trials (RCTs, case-control trials and cohort studies). In concurrence with Colman and Rubin,^16^ both free text and medical subject headings of this study included the following alternatives for fecal microbiota transplant: “fecal transplant,” “fecal transfusion,” “fecal implantation,” “fecal implant,” “fecal instillation,” “fecal donor,” “fecal enema,” “fecal reconstitution,” “fecal infusion,” “fecal therapy,” “fecal bacteriotherapy, “faecal transplant,” “faecal transfusion,” “faecal implantation,” “faecal implant,” “faecal instillation,” “faecal donor,” “faecal enema,” “faecal reconstitution,” “faecal infusion,” “faecal therapy,” “faecal bacteriotherapy, “microbiota transplant,” “microbiota transfusion,” “microbiota implantation,” “microbiota implant,” “microbiota instillation,” “microbiota donor,” “microbiota enema,” “microbiota reconstitution,” “microbiota infusion,” “microbiota therapy,” “microbiota bacteriotherapy,” “microflora transplant,” “microflora transfusion,” “microflora implantation,” “microflora implant,” “microflora instillation,” “microflora donor,” “microflora enema,” “microflora reconstitution,” “microflora infusion,” “microflora therapy,” “microflora bacteriotherapy,” “feces transplant,” “feces transfusion,” “feces implantation,” “feces implant,” “feces instillation,” “feces donor,” “feces enema,” “feces reconstitution,” “feces infusion,” “feces therapy,” “feces bacteriotherapy,” “faeces transplant,” “faeces transfusion,” “faeces implantation,” “faeces implant,” “faeces instillation,” “faeces donor,” “faeces enema,” “faeces reconstitution,” “faeces infusion,” “faeces therapy,” “faeces bacteriotherapy,” “stool transplant,” “stool transfusion,” “stool implantation,” “stool implant,” “stool instillation,” “stool donor,” “stool enema,” “stool reconstitution,” “stool infusion,” “stool therapy,” “stool bacteriotherapy,” “flora transplant,” “flora transfusion,” “flora implantation,” “flora implant,” “flora instillation,” “flora donor,” “flora enema,” “flora reconstitution,” “flora infusion,” “flora therapy,” and “flora bacteriotherapy.” The results were then combined using the set operator “AND” with studies identified by varied UC descriptor terms: “ulcerative colitis,” “inflammatory bowel disease,” “colitis,” “ileitis,” “IBD,” and “UC.” We also manually searched proceedings from major international conferences, including the American College of Gastroenterology, Digestive Disease Week, Advances in IBD, United European Gastroenterology Week, Asia Pacific Digestive Week, Congresses of Gastroenterology China and Chinese Congresses of Digestive Diseases from 2010 up to and including September 2015. Additional studies were identified by manually searching the references of articles retrieved from the computerized databases and relevant review articles.

### Study Selection and Extraction

Eligibility criteria were determined a priori by the study authors. FMT was defined as administration of a suspension of donor feces (either fresh or frozen) into the gastrointestinal tract for UC treatment. UC was defined by the researchers in studies according to laboratory confirmation, endoscopic evidence and/or clinical symptoms. Efficacy of FMT was assessed by clinical remission. The primary outcome was clinical remission of UC, defined as Mayo score ≤2,^9,11,23^ or pediatric UC activity index <10.^10,24^ Studies without reported clinical endpoints were excluded. If the study included patients with infections before FMT, they were excluded.

The studies were imported into a bibliographic database to automatically exclude duplicates. Titles, abstracts, and articles were reviewed and assessed by 2 independent reviewers (Sun and Li) based on the eligibility criteria. Data extraction from selected publication used a standardized pretested form. A third party compared the forms of data extraction (Xu and Lu). Any disagreements were corrected by consensus. Demographic data (average age, number of men), pre-FMT therapy, transplantation procedures (route of instillation, FMT dose, numbers of infusions), choice of donor, clinical resolution, adverse events, and duration of follow-up were retrieved. If certain data points were not reported, we contacted with the authors by and obtained the detailed missing data.

### Methodology Quality Appraisal

Two authors independently assessed the studies selected for inclusion for methodological quality using 3 methods. The methodological quality of cohort studies was assessed by the National Institute of Clinical Excellence (NICE) quality assessment in keeping with the previous literature^25^ (Table 1). The quality of 1 prospective case control study was assessed by the Newcastle-Ottawa Quality Assessment Scale for case-control studies that comprised 3 separate parts (selection, comparability, and exposure).^20^ For the 2 RCTs, quality was assessed by a modification of the Cochrane approach to determining risk of bias.^26^

**TABLE 1:**
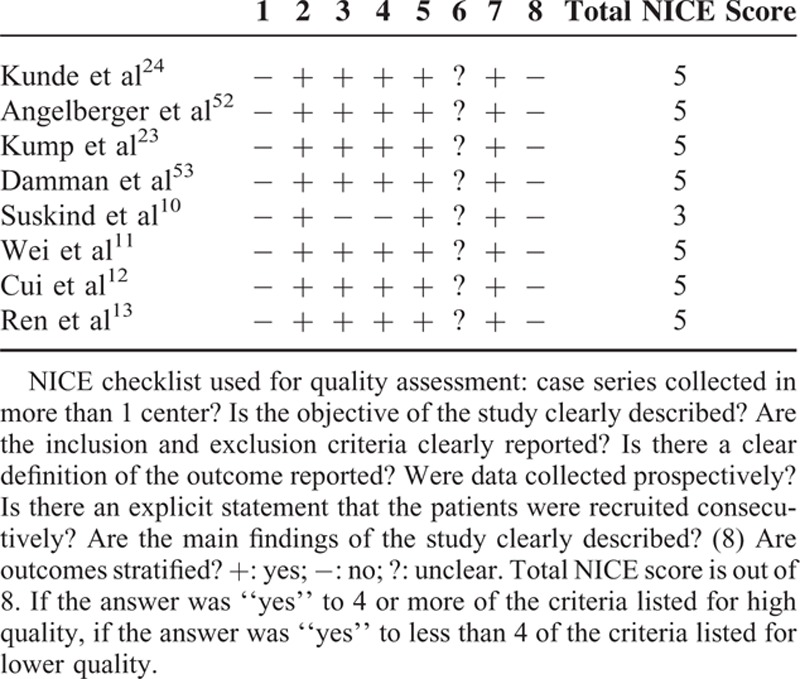
Quality Assessment of Cohort Studies According to the NICE Checklist

### Data Analysis

The overall meta-analysis included the clinical remission rates obtained from 9 cohort studies and from the FMT experimental arm of 2 randomized clinical studies and 1 case control study in keeping with the previous literature.^27^ We used a fixed-effects model assessing the pooled estimate of clinical remission in the meta-analysis with OpenMeta[Analyst]. Meta-Analyst software (version Beta 3.13; Tufts Medical Center, Boston, MA) was used to construct the funnel plot.^28^

Statistical heterogeneity for each meta-analysis was assessed using the Cochran *Q* test (χ^2^) and *I*^2^ method. In the *Q* test a *P* value of >0.1 was not deemed as statistically significant, it showed that the study was not heterogeneous, and hence we used fixed-effects models; otherwise, we used random-effects models. The *I*^2^ method was used to assess the degree of heterogeneity (a score discrimination of 0–40%, 30–60%, 50–90%, and 75–100% was consistent with low, moderate, substantial, and considerable heterogeneity, respectively).^29^ Some outcomes were not analyzed but presented in a descriptive way.

## RESULTS

### Study Selection and Included Studies

Our study search yielded 913 potentially relevant studies. We excluded 141 duplicates and 772 studies based on title and abstract screening. Thirty-four studies were retrieved in full text or abstract. Eleven studies (2 RCTs, 1 open-label case-control study, and 8 cohort studies) met the eligibility criteria. Of the 11 studies, 10 were in full text and 1 was an abstract, and all of them included UC patients for whom there were no infections before FMT. With the exception of 1 study that reported mixed patients (11 UC patients and 3 patients with Crohn disease)^11^ (Table 2), all other studies only included UC patients.

**TABLE 2:**
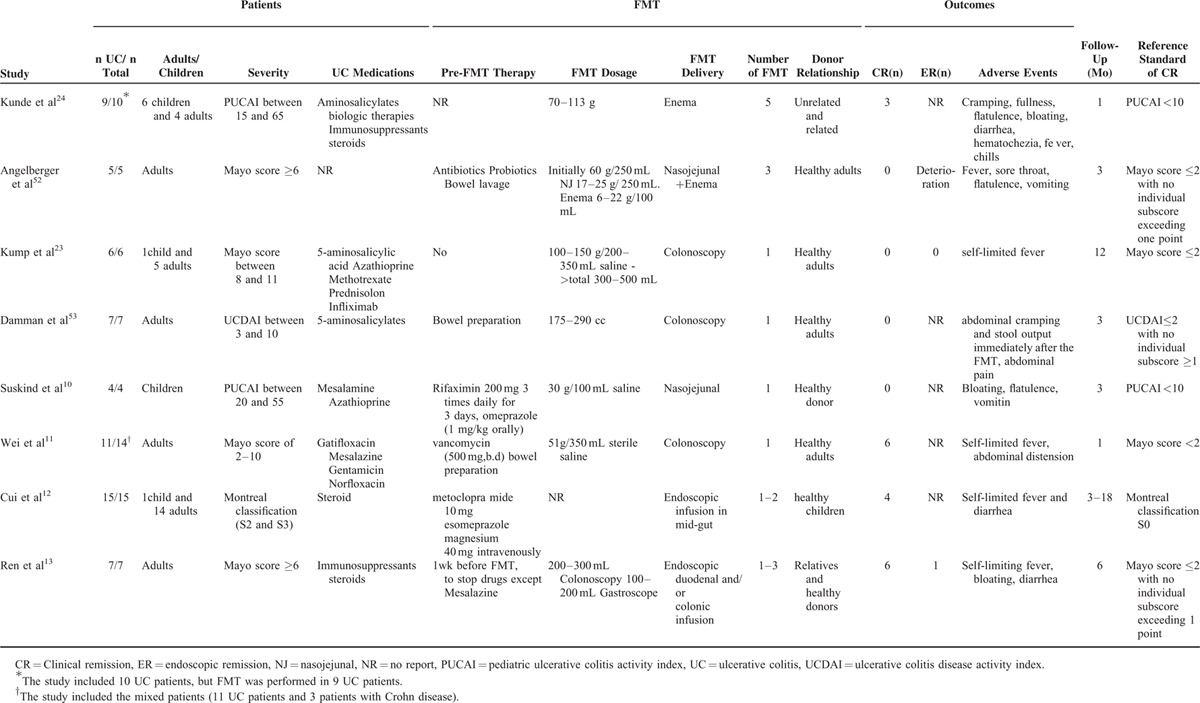
Noncontrol Cohort Studies of FMT for the Management of UC

Among the 23 excluded studies, 10 studies reported UC patients with *C difficile* infections;^30–39^ 9 were case reports of UC;^8,40–47^ 2 studies with the unclear definition of UC;^48,49^ and 2 studies did not clarify the primary clinical outcome.^50,51^ We could not obtain more detailed data about the primary clinical outcome from the authors in 2 studies,^50,51^ because the corresponding authors’ email addresses were not provided with the reports.

### Methodological Quality of Included Studies

A modification of the Cochrane approach was used to determine risk of bias of 2 RCTs.^14,15^ Adequate sequence generation and allocation concealment were not described clearly in 1 study.^15^ Reviewers determined that 2 RCTs had clear description of the blinding, incomplete outcome data, free of selective reporting, and free of other bias (Table 3). The assessment score of 1 open-label case-control study^9^ was 6-stars using the Newcastle-Ottawa Quality Assessment Scale. Among 6 stars, 3 stars were from selection (the case definition was adequate, consecutive representativeness, definition of controls), 1 star was from comparability (study controls for any additional factor), and 2 stars were from exposure (ascertainment of exposure, same method of ascertainment for cases and controls). The NICE quality assessment was used to evaluate 8 cohort studies. None of the 8 studies met all the criteria (Table 1). None of the studies were multicenter trials that recruited consecutively or stratified the outcomes. All of the studies were prospective and had clear objectives and inclusion/exclusion criteria.^10–13,23,24,52,53^ Among the 8 cohort studies, the NICE total scores of 7 studies were ≥4, but no study had a maximum NICE total score of 8. Seven studies were considered “high-quality,” while 1 study was classified as “lower quality” (Table 1).

**TABLE 3:**
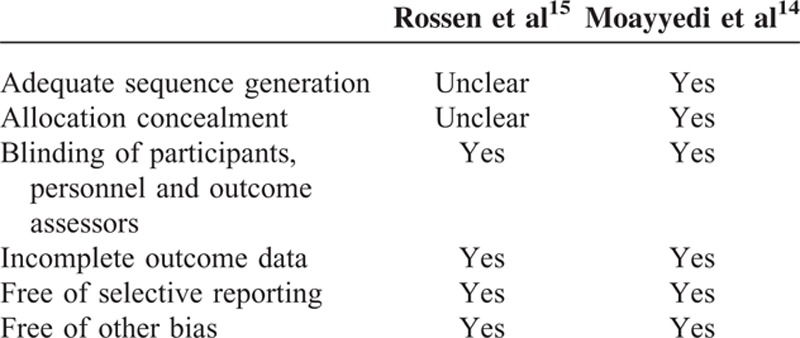
Quality Assessment of Randomized Controlled Trials

### Patients’ Demographics

Eleven studies yielded 133 UC patients with FMT (64 cases in noncontrol cohort studies, 61 cases in 2 RCTs, and 8 cases in an open-label case-control study). Studies included both pediatric and/or adult patients. Among the 133 patients included in the review, 27 (20.3%) were described as having moderate/severe disease, 100 (75.2%) as having mild or mild/moderate disease (Tables 2 and 4), and 6 cases (4.5%) where UC was active (Table 2). Duration of follow-up of patients ranged from 1 month to 18 months with median 3.7 months in 12 studies (Tables 2 and 4).

**TABLE 4:**
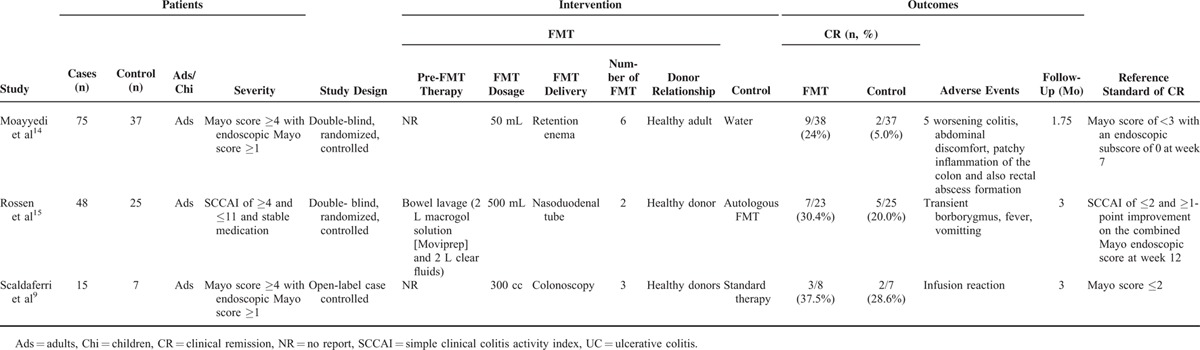
Clinical Control Trials of FMT for the Management of UC

### Fecal Microbiota Transplant of Methodological Characteristics

#### Data From RCTs

Two small double-blind, randomized (1:1), controlled trials with moderate risk of bias reported use of FMT for patients with mild-to-moderate UC.^14,15^ In the first study,^14^ 75 patients (Mayo Clinic score ≥4 with an endoscopic Mayo Clinic score ≥1) received weekly FMT or placebo (water) via retention enema for 6 weeks. Researchers and patients were blinded to the treatment allocation. The primary endpoint was clinical remission, defined as Mayo Score of <3 with an endoscopic subscore of 0 at week 7. Six donors (healthy adults) were included in the study, with the majority of subjects receiving FMT from 2 donors (A and B). The Data Monitoring and Safety Committee (DSMB) advised that the trial should be discontinued for futility because the primary end point was unlikely to be achieved as specified in the protocol. At the conclusion of the study, the authors found that patients who received FMT achieved better CR than those receiving placebo (9/38 [24%] vs. 2/37 [5%]; *P* = 0.03). In the second study,^15^ 50 UC patients (simple clinical colitis activity index (SCCAI) of ≥4 and ≤11 and stable medication) were treated with either donor stool or autologous FMT (infusion of their own stool) delivered via nasoduodenal tube at baseline and again 3 weeks later. Participants and trial members were blinded to the treatment allocation. Only 37 subjects completed assessment for the primary endpoint, which was also CR defined as SCCAI of ≤2 in combination with ≥1-point improvement on the combined Mayo endoscopic score at week 12. This study was also terminated at interim analysis by the DSMB because of futility. There was no difference in clinical and endoscopic remission between the 2 groups in either the intention-to-treat or per-protocol analyses.

#### Data From an Open-Label Case-Control Study

An open-label case-control study was included in the review.^9^ Fifteen patients (Mayo score ≥4 with an endoscopic Mayo score ≥1) were treated with either FMT (8 patients) or standard therapy (7 patients). Enrolled patients underwent colonoscopy and received 3 administration of FMT using 200 cc of fecal slurry from a healthy donor. The CR was the second endpoint defined as partial Mayo score ≤2 with no subscore ≥1 at 2 weeks. Overall, the authors found that the CR were not significantly different between FMT and standard therapy (3/8 [37.5%] vs. 2/7 [28.6%]).

#### Data From Noncontrol Cohort Studies

All 8 studies in this category were prospective, noncontrol cohort design. Incomplete data were reported for the donors, preparation, and administration of FMT (Table 2). Eight studies utilized unrelated healthy donors, including 1 study that used healthy children,^12^ 4 studies used healthy adults,^11,23,52,53^ and 1 study did not describe the ages of donors.^10^ Two studies used related and unrelated donors.^13,24^ The delivery method included colonic delivery (including enema administration and/or colonoscopic instillation) (n = 4),^11,23,24,53^ upper gastrointestinal delivery (including nasogastric/nasojejunal and gastroscopic instillation) (n = 2),^10,12^ and the combination of colonic and upper gastrointestinal delivery (n = 2).^13,52^ The FMT dosage and number of FMT were also variable. The FMT dosage was calculated by the volume of the FMT suspension (containing sterile water or saline) or the weight of stool. The number of FMT ranged from 1 to 6 times among 8 studies.

#### Meta-Analysis

Eight noncontrol cohort studies and the treatment arms of the clinical control trials were included in a meta-analysis. The pooled proportion of patients who achieved CR was 30.4% (95% CI 22.6–39.4%) (Figure 2). There was low heterogeneity (Cochran Q test, *P* = 0.139; *I*^2^ = 33%) (Figure 2).

**FIGURE 1:**
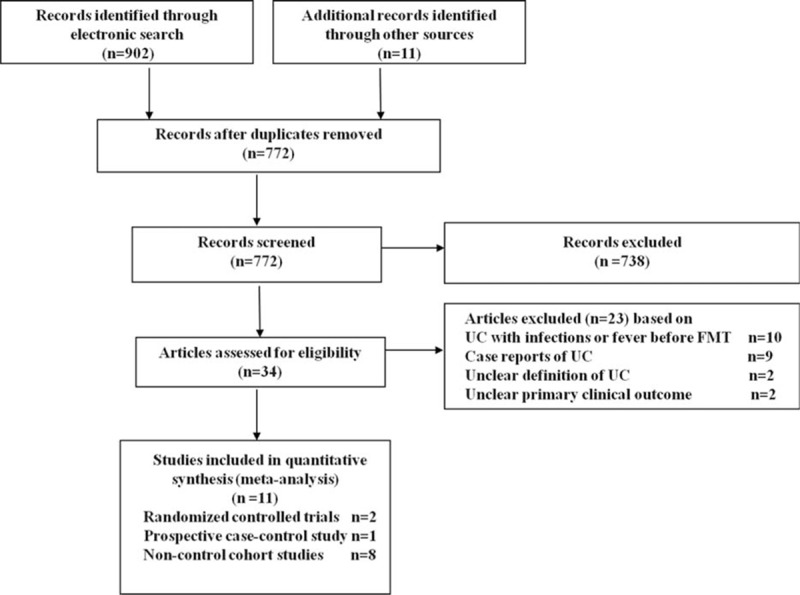
Summary of evidence search and selection.

**FIGURE 2:**
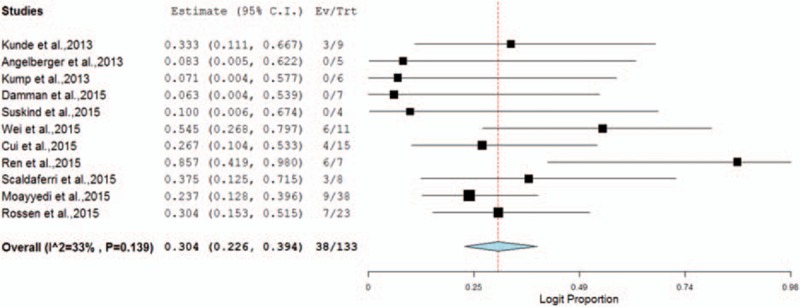
Forest plot of the clincal remission (CR) in all studies.

### Subgroup Analyses

**FIGURE 4:**
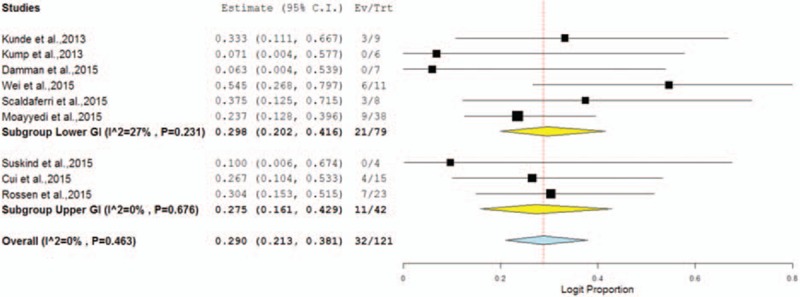
Subgroup forest plot of the clinical remission (CR) in different delivery routes.

Our first subgroup analysis compared the efficacy of upper gastrointestinal delivery (nasogastric/nasojejunal tube and gastroscopy) versus lower gastrointestinal delivery (colonoscopy and/or enema). Two studies with the combination of upper and lower gastrointestinal delivery were excluded from this analysis.^13,52^ The rate of CR in patients with the upper gastrointestinal delivery was 27.5% (95% CI 16.1–42.9%) with low heterogeneity between studies (Cochran *Q* test, *P* = 0.676; *I*^2^ = 0%). Six studies used lower gastrointestinal delivery. The rate of clinical remission was 29.8% (95% CI 20.2–41.6%) with low heterogeneity between studies (Cochran *Q* test, *P* = 0.231; *I*^2^ = 27%) (Figure 4 and Table 5).

**TABLE 5:**
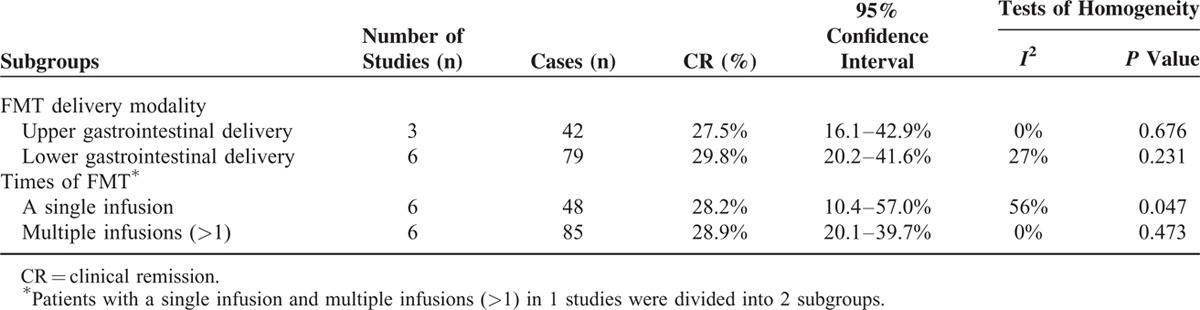
Subgroup Analysis for FMT in UC Patients

**FIGURE 3:**
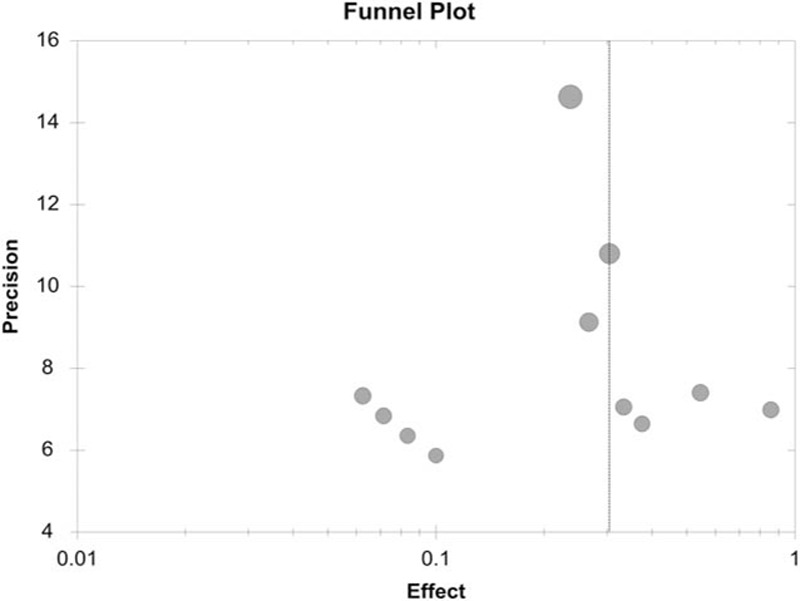
Funnel plot of the meta-analysis (all studies).

Our second subgroup analysis compared the efficacy of a single infusion versus multiple infusions (>1) of FMT. When studies had mixed a single infusion and multiple infusions (>1), we were able to divide and analyze individual cases, as their raw data was available.^13^ However if the patients were administered the second FMT due to no efficacy of the first FMT, those were defined as cases with failed a single infusion.^12^ The rate of CR in patients with a single infusion was 28.2% (95% CI 10.4–57.0%) with moderate heterogeneity between studies (Cochran *Q* test, *P* = 0.047; *I*^2^ = 56%). A total of 85 subjects in 6 studies received multiple infusions (>1). The rate of CR was 28.9% (95% CI 20.1–39.7%) with low heterogeneity between studies (Cochran *Q* test, *P* = 0.473; *I*^2^ = 0%) (Figure 3 and Table 5).

**FIGURE 5:**
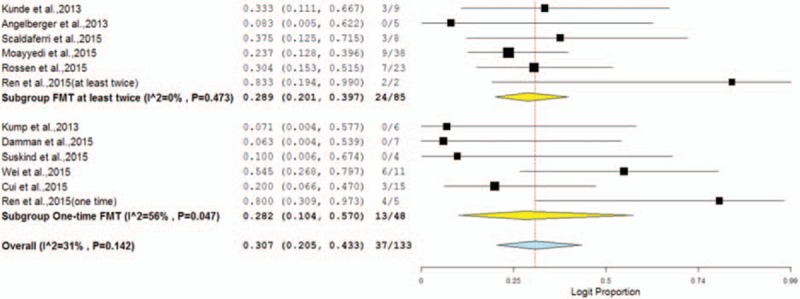
Subgroup forest plot of the clinical remission (CR) in different infusions.

### Sensitivity Analysis

We excluded the open-label case-control study that was an abstract^9^ and the study that was categorized as “lower quality”;^10^ and we performed another meta-analysis including other studies, and the CR was 30.4% (95% CI 22.3–39.9%) with moderate heterogeneity between studies (Cochran *Q* test, *P* = 0.087; *I*^2^ = 43%). The results were similar to the results of the meta-analysis of all studies.

### Adverse Events

Most of studies showed that FMT was safe (Tables 2 and 4). All of studies reported mild adverse events (including self-limiting fever, abdominal discomfort, abdominal pain, bloating, diarrhea, and vomiting). Two patients’ deterioration of UC was observed 4 weeks after FMT in a study.^52^ In a clinical randomized control study,^14^ 3 patients with FMT suffered adverse events. Two patients in the FMT group developed patchy inflammation of the colon and also rectal abscess formation, which was resolved by antibiotic therapy. One patient with worsening abdominal discomfort tested positive for *C difficile* toxin after the study.

## DISCUSSION

To date, this is the largest systematic review and first meta-analysis on FMT in UC patients without infections. We identified 2 RCTs, 1 open-label case-control study, and 8 noncontrol cohort studies about the efficacy and safety of FMT in UC patients. According to the 11 studies (8 noncontrol cohort studies and 3 treatment arms of the clinical control trials) in our review, the overall efficacy of FMT was 30.4% (95% CI 22.6–39.4%) in achieving CR, which was significantly higher than the CR of 22% (95% CI 10.4–40.8%) reported by Colman and Rubin.^16^ Because few studies were available, Colman and Rubin performed a subgroup analysis with a small sample (including 27 UC cases with FMT in 4 noncontrol cohort studies) and the confidence interval of clinical remission was distinct.

It is worth noting that FMT is not nearly as effective in UC as it is in CDI. In a systematic review and meta-analysis, the efficacy of FMT in patients with *C difficile* was significantly high at 89.% (95% CI 84.0–93.3%).^25^ These rates are substantially higher than the 30% to 80% CR rates typically reported in various medical therapies for CDI, although direct comparison of such different studies cannot be done with confidence.^54^ CDI occurs as a result of outstanding disruption of the indigenous gut microbiota by antibiotics,^55^ while UC is a complicated disease with a complex pathologic interplay among immunologic, genetic, environmental, and gut microbial factors. Manipulating the gut microbiota might be an important treatment approach, but not the only 1 strategy for UC. The use of probiotics is the most common approach used by researchers to manipulate microbiota in UC patients. Based on the clinical trial evidence available to date, only *Escherichia coli* Nissle and probiotic mix VSL#3 appear to be effective in the management of UC.^56^ The CR rates after using *Escherichia coli* Nissle^57^ and probiotic mix VSL#3^58^ were 68% and 49.4%, respectively. Based on limited approaches to manipulate the microbiota in UC patients, FMT with moderate efficacy (30.4% CR rate) might be an alternative approach.

To date, only 2 high-quality RCTs have been published,^14,15^ both of which avoid methodological flaws in previous case series and cohort studies. However, results from these 2 RCTs were contradictory because of several differences in their trial design (Table 4). Moayyedi et al^14^ administered 6 FMT infusions via the lower gastrointestinal tract, whereas Rossen et al^15^ administered 2 FMT infusions via the upper gastrointestinal tract. The delivery routes and number of FMT infusions are critical to the success of FMT, which might affect the overall results. Therefore, we performed subgroup meta-analyses to detect the differences in delivery routes and number of FMT infusions.

In examining subgroup meta-analyses alone in our review, we found significant differences in CR between lower and upper gastrointestinal delivery (29.8% vs. 27.5%, respectively). This result was consistent with that of a RCT in patients with *C difficile* infection,^59^ which included 20 patients with *C difficile* infection compared with colonoscopic and nasogastric tube administration of FMT. Those authors found that nasogastric tube administration appeared to be as effective as colonoscopic administration. However, Kassam et al^25^ and Cammarota et al^60^ reported results that favored FMT by colonoscopy or enema in patients with *C difficile* infection. Some researchers posit that the upper gastrointestinal route might destroy the active constituent of FMT and render it ineffective by the time it reaches the diseased colon; for example, bacteroidetes might be destroyed by gastric acid.^18^ However, other authors have argued that many spore-forming firmicutes require transit through the upper gastrointestinal tract in order to be effective.^18,25^ Since most patients with extensive UC often have a difficult time retaining the infused suspension after FMT via colonoscopy or enema, lower gastrointestinal delivery is suboptimal in this subpopulation of patients.^12^ In light of these observations, our review identified some researchers who used different ways to decrease the destruction of the active constituent of FMT by delivering the fecal microbiota into mid-gut through endoscope^12^ or administering drugs to promote motility of the transplanted fecal microbiota into the colon and to inhibit the secretion of gastric acid.^10,12^ However, additional high-quality research is needed for further validation of these observations.

In the review, our data found that multiple infusions (>1) had been used in many studies (Tables 2 and 4); thus, we divided patients into subgroups on the basis of the number of infusion of FMT. Patients in whom FMT had been administered multiple times (>1), the rate of clinical remission was 28.9%, while the rate of CR in patients with a single infusion was 28.2%. Although no study reported the efficacy of the number of times and frequency of infusions in the UC patients, the idea is widely recognized that patients with long-standing disease may require several infusions of feces to maintain the infused microbiota in recipients after transplantation stabilized.^52^ However, better designed studies are needed to investigate this procedural aspect.

Nonetheless, the results from subgroup analyzes are mildly heterogeneous, which considerably limits the applicability of the conclusions.

Admittedly, our review has some methodological and theoretical limitations. First, in the meta-analysis of all included studies, a lower-quality study^10^ and a study in an abstract format^9^ were included. Even with sensitivity analysis that provides a similar result, the current results require verification of additional well-designed RCTs and enough power. Second, publication bias was a concern. Because most included studies were noncontrol cohort studies, the authors might have had a desire to publish a series of successfully treated patients, while some clinical failures might have been excluded in the reports that may result in preferential reporting of successful cases. In addition, definitions of variables associated with FMT program were not standard among studies, and publications often did not report data on these variables (e.g., FMT dosage, pre-FMT therapy) (Tables 2 and 4).

Despite these limitations, these data suggest that FMT may be an efficacious and safe alternative therapy for UC, at least when the standard therapy has failed or is unacceptable to the UC patients. The positive findings in our review need to be confirmed and supported by additional well-designed randomized, double-blinded, controlled trials with enough samples. There is an urgent need to develop guidelines to standardize the process of FMT and FMT indication.
